# Case Report: Membranous nephropathy associated with mantle cell lymphoma: a rare case

**DOI:** 10.3389/fmed.2025.1627010

**Published:** 2025-08-15

**Authors:** Mian Ren, Luxi Cao, Quanquan Shen, Bin Zhu, Juan Wu

**Affiliations:** Department of Nephrology, Zhejiang Provincial People's Hospital, Affiliated People's Hospital, Urology and Nephrology Center, Hangzhou Medical College, Hangzhou, China

**Keywords:** membranous nephropathy, mantle cell lymphoma, nephrotic syndrome, secondary nephropathy, non-Hodgkin lymphoma

## Abstract

Mantle cell lymphoma (MCL) is a rare, aggressive non-Hodgkin lymphoma (NHL) that can affect the kidneys. In this research, we described a very rare case of secondary membranous nephropathy (MN) associated with MCL. A 67-year-old man manifested with impaired renal function and nephrotic syndrome (NS). Kidney biopsy revealed atypical MN and MCL cell infiltration. After eight rounds of modified R-CHOP therapy, which includes rituximab, cyclophosphamide, doxorubicin, vincristine, and prednisolone, the patient achieved complete remission, accompanied by improved renal function and a reduction in proteinuria. This case demonstrates the precise diagnosis and treatment of the primary disease, which can lead to favorable outcomes.

## Introduction

Mantle cell lymphoma (MCL) is a mature B-cell non-Hodgkin lymphoma (NHL) that accounts for approximately 5 to 7% of all lymphoma cases (1). The median age at diagnosis is between 60 and 70 years, with a higher prevalence in men than in women (1). MCL is typically diagnosed based on morphologic features and immunophenotyping. The neoplastic cells are small-to-medium lymphocytes expressing CD20, CD5, and cyclin D1. The cells are negative for CD10 and Bcl-6 and usually do not express CD23 (2).

Membranous nephropathy (MN) is a common glomerular disease. It is characterized histomorphologically by the presence of immune deposits in the subepithelial space of the glomerular filtration barrier, accompanied by diffuse thickening of the basement membrane (3, 4). The clinical manifestation of MN is nephrotic range proteinuria with edema (3, 4). The etiology and pathogenesis of MN are not fully understood. The primary MN (PMN), which is also called idiopathic MN (IMN), accounts for 70% of all MN (5). In PMN, circulating autoantibodies, mostly of the IgG4 subclass [e.g., anti-phospholipase A2 receptor (anti-PLA2R) and anti-thrombospondin type-1 domain-containing 7A (THSD7A)], bind to endogenous antigens on podocytes and are assumed to be the first step of pathophysiological changes (5, 6). In secondary MN, antibodies might bind to neoantigens or planted antigens to cause the following kidney lesions (3).

There is very limited literature on kidney lesions associated with MCL. Renal involvement in MCL patients can exhibit diverse histopathological characteristics, and damage resulting from direct tumor infiltration is rarely identified as the underlying cause, as observed in a cohort of patients with NHLs (7). In this research, we demonstrate a rare case of MN secondary to MCL.

## Case presentation

**Figure 1:**
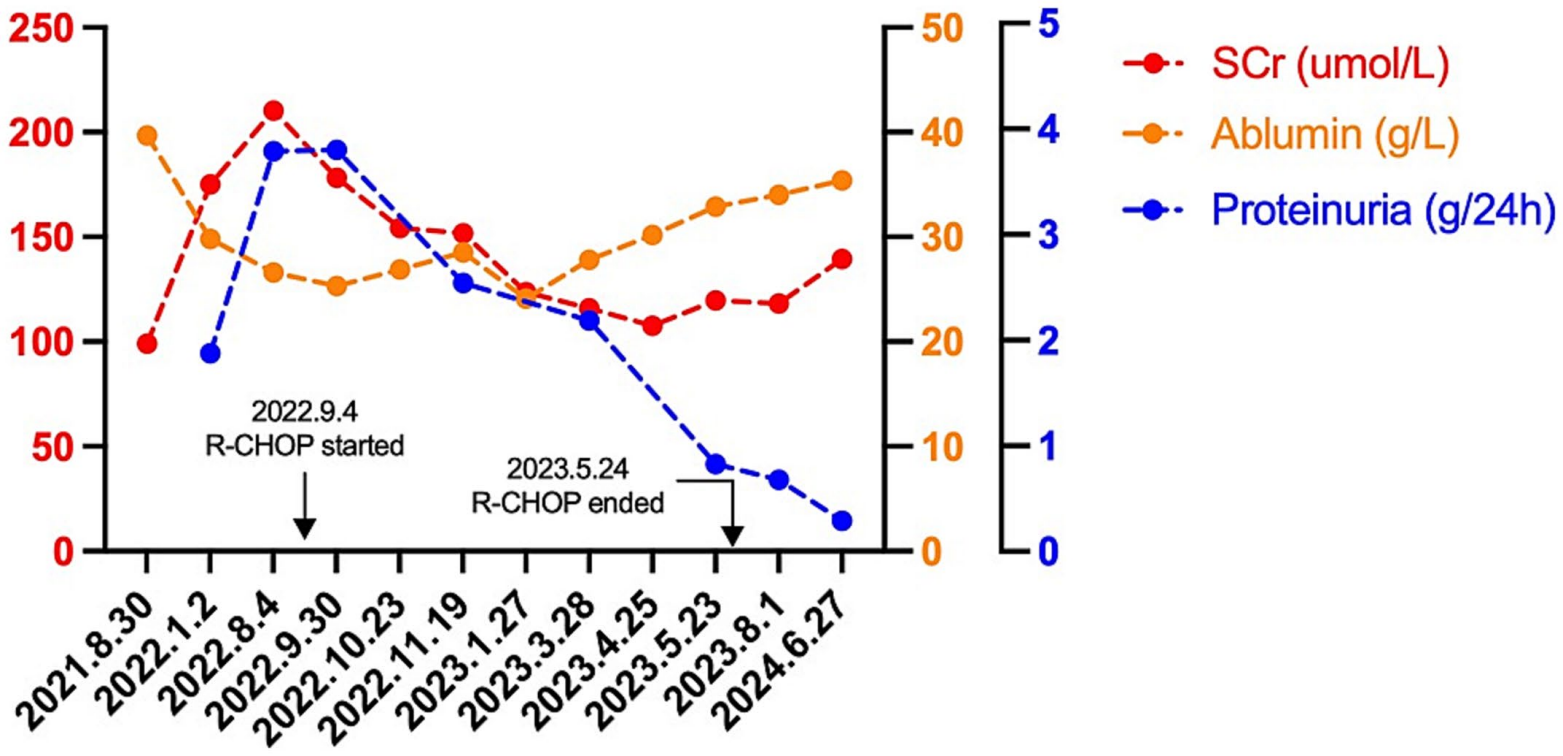
The clinical course of the case. R-CHOP indicates rituximab (375 mg/m^2^), cyclophosphamide (600 mg/m^2^), doxorubicin (60 mg/m^2^), vincristine (1.4 mg/m^2^), and prednisolone (100 mg/d × 5d).

A 67-year-old Chinese man presented to our department with a history of foamy urine and lower extremity edema for more than 10 months. Upon admission, physical examination revealed a blood pressure of 118/85 mmHg, a pulse of 95/min, a temperature of 36.6°C, a respiratory rate of 18/min, a height of 170 cm, and a weight of 72.3 kg. On physical examination, the patient showed slight edema of both lower limbs and palpable enlargement of peripheral lymph nodes, which included both cervical lymph nodes, occipital nodes, supraclavicular lymph nodes, axillary lymph nodes, and inguinal lymph nodes. Splenomegaly was also detected. Laboratory investigations revealed an elevated serum creatinine (SCr) level of 210.4 μmol/L (normal range: 57–111 μmol/L, corresponding to an estimated glomerular filtration rate (eGFR) of 27.36 mL/min/1.73 m^2^, as calculated by the CKD-EPI equation) and decreased serum albumin level of 26.6 g/L. Urinalysis showed proteinuria and microscopic hematuria (579 RBCs/μl). The 24-h proteinuria was 3.8 g (total urine volume: 1650 mL) (Figure 1). His blood leukocyte count was 6.85 × 10^9^/L, hemoglobin was 85 g/L (normocytic normochromic anemia), and platelet count was 159 × 10^9^/L. Serum globulin was increased to 51.2 g/L. Elevated IgG of 33.9 g/L and IgA of 8.26 g/L were observed. Complement 3 was 0.85 g/L, which was within the normal range, while Complement 4 was slightly decreased at 0.14 g/L. Antinuclear antibodies were positive at a dilution of 1:100 and could not be further typed. Anti-neutrophil cytoplasmic autoantibodies (ANCAs), anti-glomerular basement membrane (GBM) antibodies, serum immunofixation electrophoresis, and anti-PLA2R antibodies were all negative. Additionally, hepatitis B, C, and HIV serology were negative. Renal ultrasound revealed two normal-sized kidneys.

**Figure 2:**
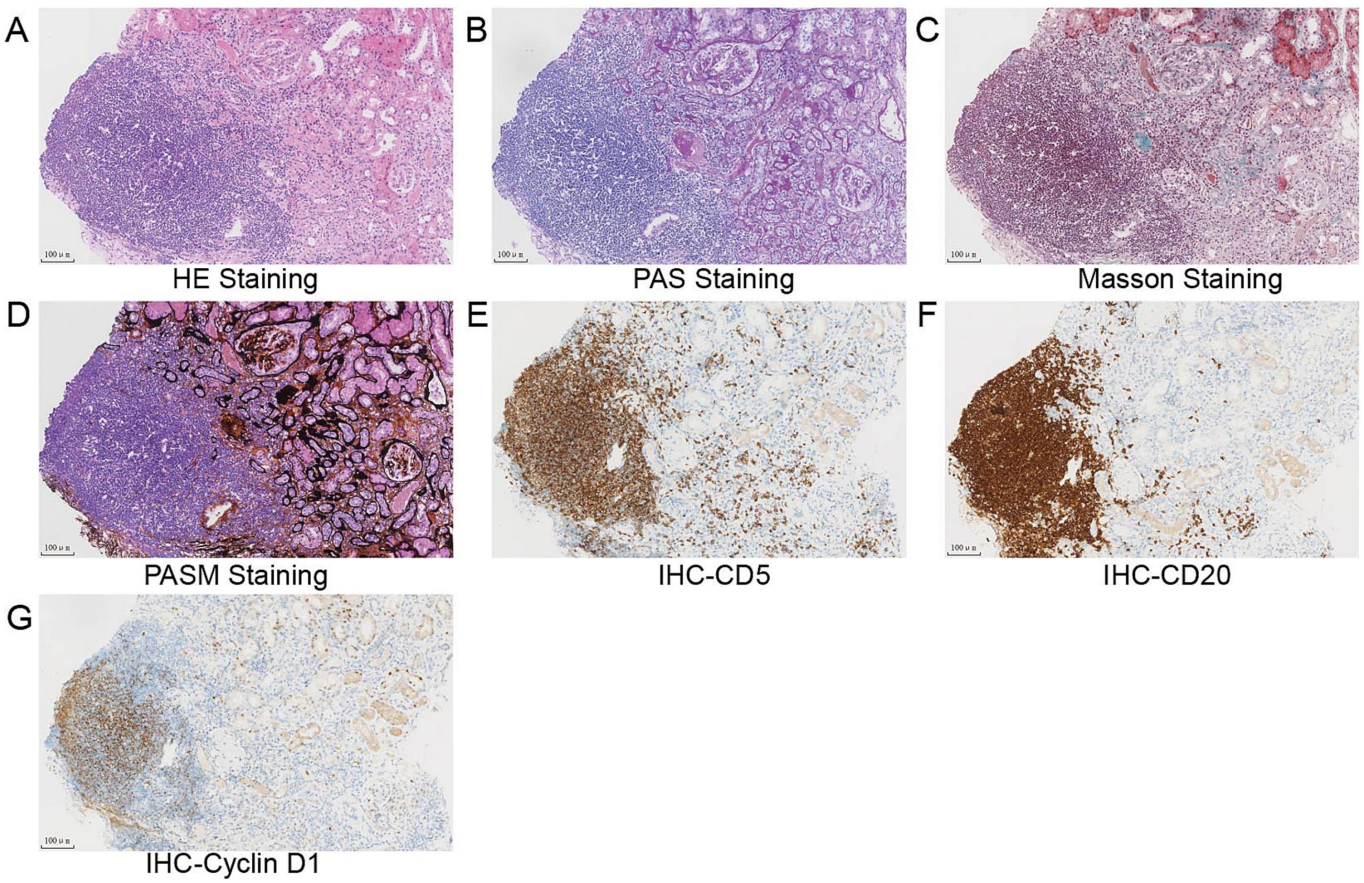
**(A–D)** Light microscopy study of the renal biopsy specimen revealed mild proliferation of mesangial cells and matrix, diffuse thickening of the glomerular basement membrane (GBM), multifocal atrophy of the tubule, and nodular lymphocytes infiltration in the tubulointerstitial **(A)** hematoxylin and eosin staining, **(B)** periodic acid–Schiff staining, **(C)** Masson trichrome staining, **(D)** periodic acid silver methenamine staining, and **(E–H)** immunohistochemical analysis revealed that lymphoblasts were strongly positive for CD5 **(E)**, CD20 **(F)**, and cyclin D1 **(G)**. Scale bars, 100 μm.

**Figure 3:**
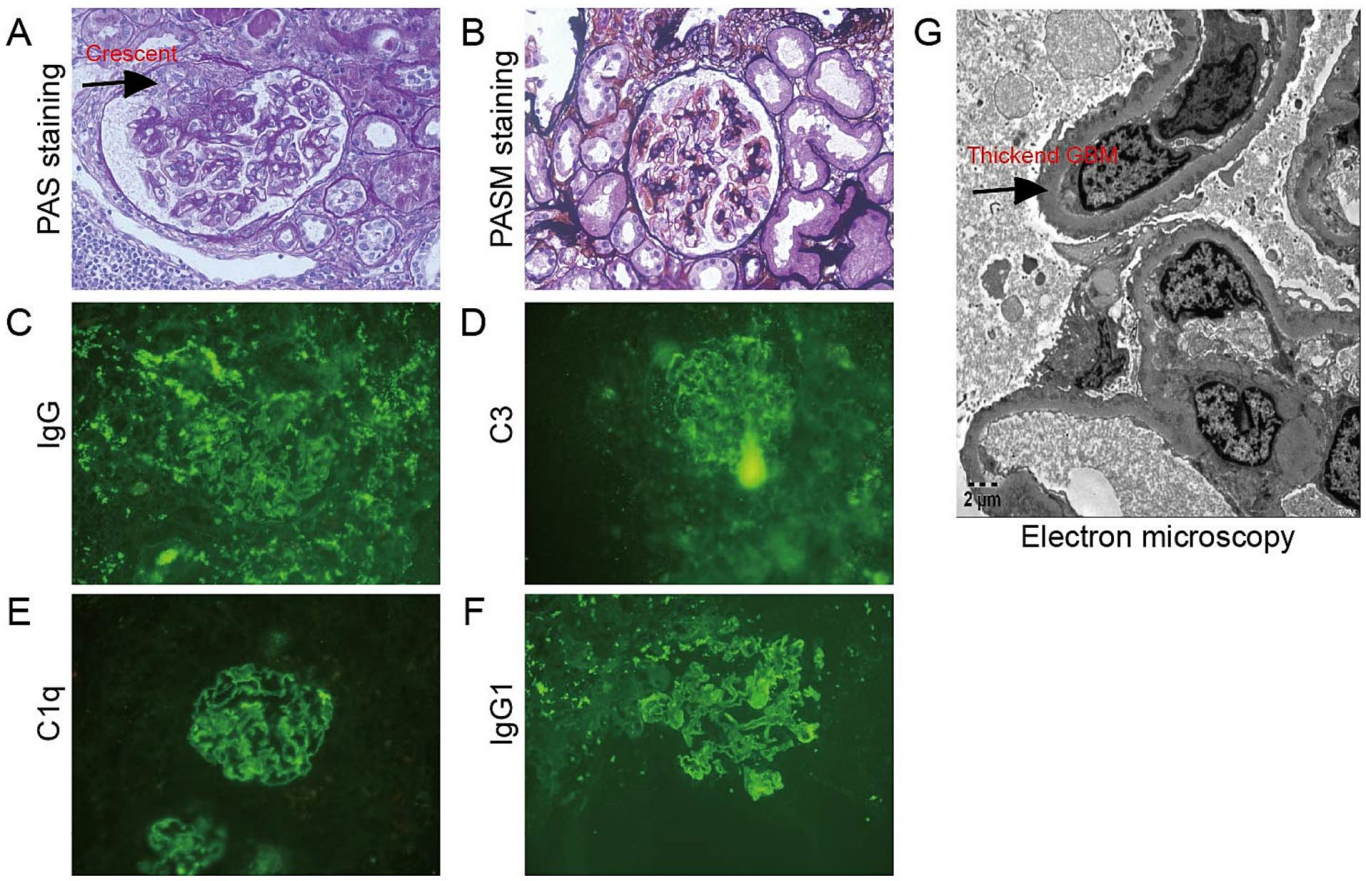
Histopathological findings in the glomerulus. **(A,B)** The glomeruli showed mild proliferation of mesangial cells and matrix, and there was one segmental cellular crescent formed (arrow) **(A)** Periodic acid–Schiff staining, ×400; **(B)** Periodic acid silver methenamine staining, ×400; **(C–F)** IgG, C3, C1q, and IgG1 granular deposits along the glomerular capillary loops direct immunofluorescence, **(C)** IgG staining; **(D)** C3 staining; **(E)** C1q staining; and **(F)** IgG1 staining; **(G)** Electron microscopy showed segmental homogenous thickening of the GBM, with thickness measuring up to 1,000 nm (arrow) (electron microscopy, ×5,000).

Flow cytometry of peripheral blood mononuclear cells (PBMCs) found 10.07% abnormal B-cell clones. Bone marrow biopsy and axillary lymph node biopsy were subsequently performed. Bone marrow flow cytometry revealed abnormal B-cell clones comprising 16.94% of cells. Immunohistopathology examination of the axillary lymph node confirmed a diagnosis of MCL, classified as Stage IVa according to the Ann Arbor staging system (8) and the Chinese guidelines for the diagnosis and treatment of MCL (9). ^(18)^F-FDG positron emission tomography/computed tomography (PET/CT) was performed to evaluate the lymph node and organ involvement (10). It revealed multiple enlarged lymph nodes of varying sizes across several regions of the body. The largest lymph node was located in the axillary region, measuring 3.9 cm × 2.1 cm, with a maximum standardized uptake value (SUVmax) of 8.0. These lymph nodes exhibited varying degrees of increased FDG metabolism, consistent with the metabolic pattern of lymphoma. Furthermore, nodular FDG-avid foci were observed in the upper limb muscles and subcutaneous tissue of the right shoulder. Splenomegaly with diffusely increased FDG uptake (SUVmax 5.8) was also noted. A renal biopsy was subsequently performed. Light microscopy revealed 20 glomeruli, including 3 with atherosclerosis and 1 with segmental cellular crescent formation. The remaining glomeruli showed mild proliferation of mesangial cells and matrix. Multifocal lymphocyte infiltration was observed in the tubulointerstitial area, with partial nodular formation (Figures 2A–D, 3A,B). The nodular regions were positive for CD20, CD5, and cyclin D1, which indicated MCL cell infiltration (Figures 2E–G). The GBM thickened diffusely. The tubule displayed multifocal atrophy. Direct immunofluorescence showed granular capillary loop staining for IgG (2+), C3 (2+), C1q (3+), IgG1 (3+), kappa (2+), and lambda (1+), but glomerular staining for IgA, IgM, C4, fibrin, HBs, HBc, IgG1, IgG2, and IgG3 were all negative (Figures 3C–F). PMN-associated antigens PLA2R and THSD7A staining were negative. Electron microscopy showed extensive foot process effacement and dense deposits in the subepithelial, basement membrane, and mesangial areas. It also indicated segmental uniform thickening of the basement membrane, reaching a thickness of up to 1,000 nm (Figure 3G). Based on these findings, the patient was diagnosed with atypical MN with MCL.

The patient’s Eastern Cooperative Oncology Group Performance Status (ECOG) performance status was 1. He received chemotherapy with modified R-CHOP, which consists of rituximab (375 mg/m^2^), cyclophosphamide (600 mg/m^2^), doxorubicin (60 mg/m^2^), vincristine (1.4 mg/m^2^), and prednisolone (100 mg, once daily for 5 days). The standard interval between cycles was 28 days. After one round of chemotherapy, the patient’s SCr level decreased to 178.3 μmol/L with an eGFR of 33.19 mL/min/1.73 m^2^ as calculated by the CKD-EPI equation. The treatment of round 2 was delayed by 28 days due to pneumonia. After the eighth round of R-CHOP therapy, the SCr was 118.2 μmol/L with an eGFR of 54.55 mL/min/1.73 m^2^. The 24-h proteinuria was 0.68 g (total urine volume: 1,650 mL) (Figure 1). Post-treatment PET/CT revealed a significant reduction in size and FDG uptake of previously involved lymph nodes. The largest lymph node of the axillary region was 1.2 cm × 0.6 cm (SUVmax 0.9). The Deauville score was 2. No enlargement or abnormal FDG uptake was observed in the spleen. The latest follow-up was 1 year post-treatment. The patient’s SCr was 139.5 μmol/L, with an eGFR of 35.4 mL/min/1.73 m^2^. The 24-h proteinuria was 0.29 g/24 h. (Figure 1).

## Discussion

MCL is a rare, aggressive lymphoma. Renal involvement of MCL has been reported previously. The histopathological features include membranoproliferative glomerulonephritis (MPGN) (11), minimal change disease (MCD) (12), ANCA-associated glomerulonephritis (13), lupus nephritis (14), and lymphoma infiltrating the kidney parenchyma. MN secondary to MCL is indeed rare.

In this study, we present the case of a 67-year-old man with newly diagnosed mantle cell lymphoma and MN secondary to MCL. Renal impairment was significantly improved by chemotherapy for MCL. It is well known that MN is the most frequent renal injury associated with solid tumors, but only a few cases have reported the association between MN and lymphoma (15, 16). As in Hodgkin’s disease, the most common renal lesion is minimal-change nephrotic syndrome (17), whereas the association of MN with non-Hodgkin lymphomas has been rarely reported (18). Nie’s study showed that renal amyloidosis and MN were the most common glomerular diseases in B-cell lymphoproliferative disorders (19). Another study found that the most common glomerular lesion is membranoproliferative GN, followed by MN in NHL and chronic lymphocytic leukemia (CLL) (7). In a multi-institutional study, the authors retrospectively reviewed patients’ kidney biopsy samples of those who had active and treated MCL (20). There were 20 patients who had active MCL at the time of biopsy, of which 14 presented with acute kidney injury, proteinuria, and/or hematuria due to MCL. Kidney pathology revealed that two patients had proliferative glomerulonephritis with monoclonal immunoglobulin deposits (PGNMID), two had C3GN, three had secondary MN, two had tubular basement membrane deposits, and two had modest lupus-like GN (20). Several case reports have described kidney involvement in MCL; however, only a few have reported MN associated with MCL (21).

In MN, a high level of serum anti-PLA2R antibodies and the presence of PLA2R antigen in the kidney are usually associated with primary forms of MN (22). THSD7A is currently believed to be used to assess PMN activity and patient prognosis (23). PLA2R and THSD7A have diagnostic significance for PMN (22, 23). For secondary MN, several new target antigens, such as neural epidermal growth factor-like 1 protein (NELL-1), exostosin 1 (EXT1), EXT2, semaphorin 3B (SEMA3B), and serine protease HTRA1, have been found in recent years (3). However, these newly found target antigens have not yet been widely applied in clinical practice. In our case, PLA2R and THSD7A were negative, indicating that the patient likely had secondary MN. Kidney immunohistochemistry (IHC) revealed positive expression of CD5, CD19, and cyclin D1, meeting the most recent MCL diagnostic criteria (9). Based on these findings, we diagnosed the patient with secondary MN due to MCL. The precise pathologic mechanisms and target antigens responsible for disease in secondary MN are less well understood. It is assumed that circulating antigens, immune complexes, or monoclonal immunoglobulins may “plant” on the subepithelial side of the GBM by virtue of size and/or charge and thereby initiate immune complex formation in the subepithelial position (24). Researchers found that RAS signaling via the Wnt1/*β*-catenin pathway, gut microbiota, oxidative stress, and inflammation were involved in MN pathogenesis (25–27). However, no study, to date, has reported a specific mechanism of MCL-induced MN. It still needs to be explored.

For PMN, all patients should receive supportive management, such as angiotensin-converting enzyme inhibitors or angiotensin-receptor blockers, blood pressure control, and sodium intake restriction (24). Based on the disease classification, PMN patients will receive different treatment strategies, such as corticosteroids and immunosuppressants. There are also some traditional Chinese medicines (e.g., Tripterygium wilfordii multiglucoside) that have been found to be effective in MN (28). However, the general treatment principle for secondary MN is to treat the underlying disease. In patients with malignancy-associated MN, treating the malignancy should be paramount (24). Older patients and those with serious coexisting conditions may not be candidates for autologous transplantation (1). In our case, the patient was elderly and had significant renal impairment at diagnosis, making him unsuitable for autologous stem cell transplantation (ASCT). According to the guidelines for the diagnosis and treatment of MCL in China (5), patients over 65 years of age, those with poor general condition, or those unsuitable for ASCT should receive non-intensive regimens such as R-CHOP, bendamustine-rituximab (B-R), or Venetoclax-Rituximab-Cyclophosphamide-Doxorubicin-Prednisone (VR-CAP). In addition to chemotherapy, several trials have investigated “chemotherapy-free” regimens as initial treatments for MCL. A study evaluating lenalidomide plus rituximab as first-line treatment reported a response rate of 92%, with a complete response rate of 64% (29). In the US, the combination of ibrutinib and rituximab was studied, achieving a complete response rate of 71% (30). Additionally, numerous treatment approaches have emerged in recent years, including targeted therapies such as BTK and BCL2 inhibitors, which have demonstrated durable responses (31, 32). Cellular therapies, such as chimeric antigen receptor (CAR) T cells and bispecific T-cell engager (BiTE) antibodies, have shown promising results (33, 34). However, it is essential to note that these pilot studies included a limited number of highly selected patients. More time and research are needed to prove the efficacy of these therapeutic approaches. In our case, the patient chose to receive R-CHOP therapy for MCL, which resulted in complete remission. His creatinine and proteinuria decreased significantly, demonstrating the treatment’s effectiveness.

## Conclusion

In summary, we reported the case of an old male patient who presented with nephrotic syndrome and impaired kidney function attributed to MCL. Renal biopsy showed atypical MN with concomitant interstitial infiltration from lymphoid cells. This case was diagnosed as MN secondary to mantle cell lymphoma. A longer follow-up is necessary to evaluate the long-term outcomes of treatment. Additionally, further research is needed to elucidate the pathogenesis of lymphoma-associated MN.

## Data Availability

The original contributions presented in the study are included in the article/supplementary material; further inquiries can be directed to the corresponding author.
